# Classification of epileptic seizures in EEG data based on iterative gated graph convolution network

**DOI:** 10.3389/fncom.2024.1454529

**Published:** 2024-08-29

**Authors:** Yue Hu, Jian Liu, Rencheng Sun, Yongqiang Yu, Yi Sui

**Affiliations:** ^1^College of Computer Science and Technology, University of Qingdao, Qingdao, China; ^2^Yunxiao Road Outpatient Department, Qingdao Stomatological Hospital, Qingdao, China

**Keywords:** seizure classification, GCN, iterative graph optimization, long-term dependencies in EEG series, imbalanced distribution

## Abstract

**Introduction:**

The automatic and precise classification of epilepsy types using electroencephalogram (EEG) data promises significant advancements in diagnosing patients with epilepsy. However, the intricate interplay among multiple electrode signals in EEG data poses challenges. Recently, Graph Convolutional Neural Networks (GCN) have shown strength in analyzing EEG data due to their capability to describe complex relationships among different EEG regions. Nevertheless, several challenges remain: (1) GCN typically rely on predefined or prior graph topologies, which may not accurately reflect the complex correlations between brain regions. (2) GCN struggle to capture the long-temporal dependencies inherent in EEG signals, limiting their ability to effectively extract temporal features.

**Methods:**

To address these challenges, we propose an innovative epileptic seizure classification model based on an Iterative Gated Graph Convolutional Network (IGGCN). For the epileptic seizure classification task, the original EEG graph structure is iteratively optimized using a multi-head attention mechanism during training, rather than relying on a static, predefined prior graph. We introduce Gated Graph Neural Networks (GGNN) to enhance the model's capacity to capture long-term dependencies in EEG series between brain regions. Additionally, Focal Loss is employed to alleviate the imbalance caused by the scarcity of epileptic EEG data.

**Results:**

Our model was evaluated on the Temple University Hospital EEG Seizure Corpus (TUSZ) for classifying four types of epileptic seizures. The results are outstanding, achieving an average F1 score of 91.5% and an average Recall of 91.8%, showing a substantial improvement over current state-of-the-art models.

**Discussion:**

Ablation experiments verified the efficacy of iterative graph optimization and gated graph convolution. The optimized graph structure significantly differs from the predefined EEG topology. Gated graph convolutions demonstrate superior performance in capturing the long-term dependencies in EEG series. Additionally, Focal Loss outperforms other commonly used loss functions in the TUSZ classification task.

## 1 Introduction

Epilepsy, a chronic non-communicable neurological disorder, significantly impacts individuals of all ages. Global statistics show that ~50 million people live with epilepsy, making it one of the most common neurological disorders worldwide. It is estimated that appropriate treatment could prevent seizures in up to 70% of these patients (World Health Organization, 2024). Electroencephalogram (EEG) signals, are acquired using an electrode placement scheme on the scalp (i.e., International 10-20 system; Homan et al., 1987) which records activity in different brain regions. EEG signals are generated by unconscious activation of the central nervous system, are objective and reliable, and is not easily controlled by subjective consciousness (Adeli and Ghosh-Dastidar, 2010). EEG signals can reveal the presence of abnormal brain activity and can be used to understand neuronal activity patterns associated with brain disorders (Nunez and Srinivasan, 2006).

However, the brain is a complex, densely connected system that operates across multiple spatial and temporal scales. Therefore, EEG signals are often highly correlated in space and time, which poses a huge challenge to EEG-based epilepsy detection and classification. Recent developments in deep learning (DL) techniques, particularly Convolutional Neural Networks (CNN) and Recurrent Neural Networks (RNN), have proven to be superior for such tasks (Li et al., 2020). DL can automatically learn discriminative features from raw EEG data rather than relying on manually engineered features, which can be time-consuming, domain-specific, and may not capture all relevant information in the data (Craik et al., 2019). A deep 13-layer CNN network was developed for the first time to classify normal, preictal, and epileptic seizure categories (Acharya et al., 2018). The advantage of the model was that it did not require feature extraction or feature selection. A fully convolutional network (a special type of CNN) was proposed to detect epilepsy, eliminating the need for manual feature extraction during data preprocessing (O'Shea et al., 2020). Incorporating prior knowledge into DL models can significantly enhance their performance. For example, a CNN that uses frequency domain EEG features as input was found to be more effective for seizure detection (Zhou et al., 2018). A shallow CNN architecture combined with wavelet packet decomposition was developed to extract EEG data features from both time and frequency domains, improving the accuracy of epilepsy seizure prediction (Zhang Y. et al., 2019).

Epileptic activity often exhibits distinctive temporal patterns in EEG recordings. To better explore the temporal characteristics of EEG signals, Long Short-Term Memory (LSTM) networks (i.e., a specialized form of RNN) have been adopted for seizure detection and classification. A two-layer LSTM network was proposed to predict epileptic seizures, and compared to CNN, the seizure prediction performance was significantly improved (Tsiouris et al., 2018). A seizure detection model based on bidirectional LSTM was designed, which helps to collaboratively infer the output using preceding and succeeding information relative to a given time (Hu et al., 2020). Leveraging the strengths of both CNN and LSTM, a CNN-LSTM network was developed to capture the spatial-temporal features of EEG signals (Shahbazi and Aghajan, 2018).

CNN excel at capturing local spatial patterns, while RNN are effective at modeling temporal dependencies in sequential data. However, they lack mechanisms to directly handle graph structures. Brain connectivity involves various interacting regions and exhibits complex behaviors. EEG networks provide a natural representation of brain connectivity, where EEG channels represent nodes and the connections between nodes represent correlations (Bullmore and Sporns, 2009; Rubinov and Sporns, 2010). CNN and RNN struggle to capture the intricate structures inherent in graph-structured EEG network data (Ahmedt-Aristizabal et al., 2021).

To address this challenge, Graph Convolutional Networks (GCN) (Scarselli et al., 2008) have been used in brain disease detection and classification. A GCN-based model was proposed for automatic neonatal epilepsy detection, where the temporal information contained in the EEG signals was treated as graph signals, and the spatial interdependence between brain regions was represented as functional connections among the EEG channels (Raeisi et al., 2022). Large-scale EEG datasets (the TUH EEG Corpus and MPI LEMON database) was used to validate the effectiveness of GCN for seizure detection (Wagh and Varatharajah, 2020). In Tang et al. (2021), EEG electrodes were represented as nodes, and two types of edges were generated based on the physical distance and cross-correlation. Based on these two graph structures, GCN were applied for seizure detection and multi-seizure type classification. In other EEG-based tasks, such as emotion recognition, GCN have demonstrated good performance. For example, a regularized graph neural network with node-wise domain adversarial training and emotion-aware distribution learning was proposed to address the cross-subject EEG variations (Zhong et al., 2020). Graph convolution and regular convolution were used together to extract features from graph input (Zhang T. et al., 2019).

Although GCN is very effective in processing graph-structured data, several challenges remain. First, the graph topology used in GCN is often based on expert knowledge, which may not accurately represent the connections between EEG channels. EEG signals vary significantly across different patients, making it difficult, if not impossible, to construct a predefined graph that is effective across a large number of patients (Lian et al., 2020). The methods for generating the prior graph structure can be summarized as follows:

• Euclidean distance is used to establish the graph structure among different EEG channels (Song et al., 2018; Zhong et al., 2020; Tang et al., 2021). Specifically, the value *a*_*ij*_ of this graph is obtained as:


$$\begin{array}{l} a_{ij}=exp\left(-\frac{dist{\left(v_{i},v_{j}\right)}^{2}}{\tau^{2}}\right) \end{array}$$

where *dist*(·, ·) represents the geodesic distance between the electrodes *v*_*i*_ and *v*_*j*_ on the brain region with coordinates (i.e., International 10-20 system), and τ is a scaling constant.

• Functional correlation is another frequently used method to generate a prior graph, which is determined by the time series correlation of the signals from two electrodes. The generalized form can be expressed as:


$$\begin{array}{l} a_{ij}=\frac{xcorr\left(x_{i},x_{j}\right)}{||x_{i}||||x_{j}||} \end{array}$$

where *x*_*i*_ and *x*_*j*_ represent the signals series of two electrodes *v*_*i*_ and *v*_*j*_, and *xcorr*(·, ·) is the cross-correlation function, such as spectral coherence (Wagh and Varatharajah, 2020), phase locking value and magnitude squared coherence (Raeisi et al., 2022), and directed transfer function (Ho and Armanfard, 2023), and among others.

The two types of prior graphs mentioned above are often used together for model input (Jin et al., 2021; Tang et al., 2021), or to generate more complex graph data through weighted summarization (Wagh and Varatharajah, 2020; Yu, 2022). However, prior graphs derived from basic feature spaces, can be noisy or incomplete, potentially failing to capture the actual topology of EEG signals at a higher level. Therefore, using a prior graph as input for different subjects may impair the performance of GCN. Researchers have explored various solutions to this problem. A dynamic graph convolutional neural network was proposed to learn the intrinsic relationship between EEG channels (Song et al., 2018; Jin et al., 2021). This approach used backpropagation (BP) to iteratively update the adjacency matrix representing the connection between EEG channels. However, this method introduced a large number of parameters that need to be learned, and also involved the setting of the learning rate. In Lian et al. (2020), for learning optimal graph structure, subgraph clustering were first used to obtain a coarse graph and then several convolutional operations were used to refine the graph structure. This approach was not end-to-end, and details of the subgraph clustering process were not provided. In this work, we introduce Iterative Deep Graph Learning (IDGL) architecture (Chen et al., 2020) to optimize the input graph. This approach jointly optimizes both the graph structure and the classification task. Our experiments show that the optimal graph has a completely different structure than the input prior graph, highlighting the limitations of using prior graphs as input.

Second, while GCN excel at identifying complex relationships among EEG channel signals, they encounter difficulties in capturing long-term sequence information. This limitation hinders their ability to recognize and utilize the long-term dependencies inherent in EEG signals, which are crucial for extracting temporal characteristics. A Gated Graph Neural Networks (GGNN) (Li et al., 2015) is an extension of a graph neural network, by updating temporal features of node signal sequences through the graph structure. GGNN was introduced to model temporal dependency in EEG signals for diagnosis of Alzheimer's disease (Klepl et al., 2023). Furthermore, to better leverage the influence of long-range neighbors, matrix multiplications in GGNN were replaced with graph diffusion convolutions (Li et al., 2017). This diffusion-based graph convolutional network, known as Diffusion Convolutional Recurrent Neural Network (DCRNN), was used to model EEG data for seizure detection and classification, and experiments showed that the approach performed well (Tang et al., 2021). However, the computation burden of DCRNN is expensive, especially when the graph is dense. In this work, we demonstrate that GGNN can achieve better performance when used with iterative graph learning.

Third, since epileptic seizure events are usually of short duration, typically have a much shorter duration compared to non-epileptic periods, epileptic EEG signal recognition is a typical imbalanced classification task. This skewed distribution can inadvertently lead models to prioritize majority classes, thereby neglecting the nuances of minority classes, which can impair the effectiveness of epilepsy detection and classification efforts. Traditional oversampling or undersampling techniques may not work well with EEG data, which has high inter-subject variability and is non-stationary over time. Recently, self-supervised learning has emerged as a promising solution to the issue of category imbalance in epilepsy datasets. This approach leverages the data itself without depending on extensive labeled datasets. For example, in Ho and Armanfard (2023), positive and negative pairs for each EEG clip were constructed and then used to train the model through contrastive strategies, i.e., minimizing intra-class distance and maximizing interclass distance. The authors reported that this self-supervised method can identify abnormal brain regions and EEG channels without requiring access to the abnormal class data during the training. A self-supervised pre-training technique using GCN was proposed to improve model performance by predicting upcoming signals (Tang et al., 2021). This approach assumed that by learning to predict the EEG signals for the next period, the model would learn task-independent representations and improve downstream seizure detection and classification. Although the above self-supervised methods are effective, they require high-quality data or rely on the model's ability to learn from its predictions, leading to increased computation and training time. To overcome this issue, we adopt Focal Loss (Lin et al., 2017) to reduce the loss contribution of easily classified samples through an adjustment factor. Our experiments show that this method is simple and effective, which outperforms the approach proposed in Tang et al. (2021).

The main contributions of this paper are as follows:

(1) **Proposed Iterative Gated Graph Convolutional Network (IGGCN) Model**. To classify multiple seizure types from EEG data, this model introduces IDGL to optimize the input graph structure, addressing the limitations of predefined prior graphs in traditional methods. By integrating a multi-head graph attention mechanism to replace the cosine similarity learning used in IDGL, the model significantly enhances its ability to capture complex spatiotemporal correlations.

(2) **The GGNN has been introduced**. Within the IDGL model, GGNN replaces the traditional GCN, enhances the model's ability to extract and understand long-term dependencies in functional connectivity of EEG signals. This integration of IDGL with GGNN, significantly improves the performance of classifying multiple seizure types.

(3) **Application of the focal loss function**. This effectively addresses the class imbalance problem in EEG classification tasks. The method is straightforward and easy to implement.

(4) **Performance validation on the Temple University Hospital EEG Seizure Corpus (TUSZ)**. The model was evaluated on the largest and most comprehensive EEG seizure dataset. Experimental results demonstrate that the model outperforms existing methods in classifying four types of seizures.

## 2 Methodology

### 2.1 Iterative deep graph learning and its improvements

The principle of IDGL (Chen et al., 2020) is to learn better graph structures based on better node embeddings while learning better node embeddings based on better graph structures. It first learns the graph structures using weighted cosine similarity as a metric function, as shown in Equation 1:


(1) $$\begin{array}{l} s_{ij}^{p}=cos\left(w_{p}⊙v_{i},w_{p}⊙v_{j}\right) \end{array}$$

where ⊙ denotes the Hadamard product, *w* is a learnable weight vector, *v*_*i*_ and *v*_*j*_ are the input feature vectors of EEG channels *i* and *j*. In order to stabilize the learning process and improve the expressive power, it is extended to a multi head version, as shown in Equation 2:


(2) $$\begin{array}{l} s_{ij}=\frac{1}{Q}\overset{Q}{\underset{p=1}{\sum }}s_{ij}^{p} \end{array}$$

IDGL iteratively updates the graph structure, and the iteration stops when the learned structure closely approximates the optimized graph. Each iteration updates the graph structure as shown in Equation 3:


(3) $$\begin{array}{l} {\tilde{A}}^{\left(m\right)}=\lambda L^{\left(0\right)}+\left(1-\lambda \right)\left\{\eta f\left(A^{\left(m\right)}\right)+\left(1-\eta \right)f\left(A^{\left(1\right)}\right)\right\} \end{array}$$

where *L*^(0)^ = *D*^(0)^^−1/2^*A*^(0)^*D*^(0)^^−1/2^ is the normalized adjacency matrix of the initial graph *A*^(0)^, *A*^(1)^ and *A*^(*m*)^ are the adjacency matrices at the 1-st and *m*-th iterations, respectively, *f*(·) denotes the normalization function, and *η* and λ are hyperparameters.

EEG signals often exhibit complex spatial-temporal correlations, that simple similarity measures, such as cosine similarity, may not effectively capture. In this work, we use a multi-head graph attention mechanism instead of the cosine similarity used in IDGL. By leveraging this multi-head graph attention mechanism (Veličković et al., 2017), the model can learn to capture intricate relationships between different regions of the brain over time, allowing for a more nuanced understanding of the data. The feature vectors *h*_*i*_ and *h*_*j*_ of adjacent EEG channels are initially augmented by a learnable weight vector *w*. Subsequently, these augmented features are concatenated, followed by the utilization of a parameterized function *α*(·) to map the resulting concatenation to a scalar value. This process yields the attention score *e*_*ij*_ of node *i* for *j*.


(4) $$\begin{array}{l} e_{ij}^{k}=\alpha \left(w_{k}⊙h_{i},w_{k}⊙h_{j}\right) \end{array}$$

This process computes attention scores for each pair of adjacent nodes, which are then normalized using *softmax* to ensure that the sum of attention scores for a node's neighbors equals 1. These normalized scores measure the similarity between nodes.


(5) $$\begin{array}{l} a_{ij}^{k}=softmax\left(e_{ij}^{k}\right)=\frac{exp\left(L\text{Re}LU\left(e_{ij}^{k}\right)\right)}{\sum_{j\in N_{i}}exp\left(L\text{Re}LU\left(e_{ij}^{k}\right)\right)} \end{array}$$

where *N*_*i*_ is the set of neighboring channels for channel *i*. Execute *k* independent attention mechanisms concurrently, each equipped with distinct weight parameters. Subsequently, the similarity matrices generated by each head are aggregated to produce the ultimate attention score matrix *A*.


(6) $$\begin{array}{l} A_{ij}=\frac{1}{K}\overset{K}{\underset{k=1}{\sum }}a_{ij}^{k} \end{array}$$

### 2.2 Gated graph neural networks

GGNN, introduced by Li et al. (2015) in 2015, incorporate Gated Recurrent Units (GRU) (Cho et al., 2014) into the message-passing phase of GCNs (Scarselli et al., 2008). This innovation enables GGNN to effectively capture the long-term dependencies among nodes within a graph structure through the gating mechanism. Additionally, the dynamic update of node states becomes feasible. By allowing nodes to share parameters, GGNN not only streamline the model by reducing its parameter count but also aid in mitigating overfitting. This parameter-sharing feature is crucial for enhancing the model's generalizability and efficiency. The process through which GGNNs propagate information is meticulously designed to ensure optimal learning and representation of graph-structured data.


(7) $$\begin{array}{l} H^{\left(0\right)}=X,H^{\left(t\right)}=\text{Propagator}\left(H^{\left(t-1\right)},A\right) \end{array}$$

where *A* ∈ ℝ^*n*×*n*^ denotes the adjacency matrix, *n* is the number of nodes in the graph, *X* is the feature matrix and *Propagator* module consists of the following parts (Equations 8–12):


(8) $$\begin{array}{l} \;a^{\left(t\right)}=A^{T}H^{\left(t-1\right)}+b \end{array}$$

(9) $$\begin{array}{l} z^{\left(t\right)}=\sigma \left(W^{z}a^{\left(t\right)}+U^{z}H^{\left(t-1\right)}\right) \end{array}$$

(10) $$\begin{array}{l} r^{\left(t\right)}=\sigma \left(W^{r}a^{\left(t\right)}+U^{r}H^{\left(t-1\right)}\right) \end{array}$$

(11) $$\begin{array}{l} \tilde{h^{\left(t\right)}}=tanh\left(W^{h}a^{\left(t\right)}+U^{h}\left(r^{\left(t\right)}⊙H^{\left(t-1\right)}\right)\right) \end{array}$$

(12) $$\begin{array}{l} H^{\left(t\right)}=\left(1-z^{\left(t\right)}\right)⊙H^{\left(t-1\right)}+z^{\left(t\right)}⊙\tilde{h^{\left(t\right)}} \end{array}$$

where *b* denotes the bias coefficient, *σ* denotes sigmoid activation function, *tanh* denotes hyperbolic tangent activation function, and ⊙ denotes Hadamard product. *z* and *r* are called the update gate and reset gate, respectively. *z* controls the forgetting of unwanted information and *r* controls the incorporation of newly generated information. *W*^*z*^, *U*^*z*^, *W*^*r*^, *U*^*r*^, *W*^*h*^, *U*^*h*^ are learnable weight matrices.

### 2.3 Joint losses

The loss function *L* of our IGGCN model is the sum of the predicting loss *L*_*pred*_ and the graph regularizing loss *L*_*G*_.

To address the class imbalance issue mentioned above, the Focal Loss function (Lin et al., 2017) was introduced as shown Equation 13. Focal Loss effectively reduces the loss of easily classified samples through an adjustment factor, enabling the model to focus more on challenging samples that are difficult to classify. Additionally, Focal Loss can enhance the model's robustness to outliers and noisy data. By decreasing the loss weight of easily classified samples, the model can better concentrate on critical, hard-to-classify samples, thereby improving overall classification performance.


(13) $$\begin{array}{l} L_{pred}\left(p_{x}\right)=-\alpha_{x}{\left(1-p_{x}\right)}^{\gamma }log\left(p_{x}\right) \end{array}$$

where *p*_*x*_ represents the predicted probability of a sample belonging to class *x*. The class weight coefficient *α* is defined as the reciprocal of the number of samples in each class, serves to adjust the weighting between easily and difficult-to-classify samples. The modulation coefficient *γ* is used to adjust the degree of penalty that the loss function applies to samples that are easily classified correctly.

For graph regularization loss *L*_*G*_, we take into account the smoothness loss (Belkin and Niyogi, 2001), degree regularization loss, and sparsity loss (Kalofolias, 2016). The smoothness loss ensures that adjacent nodes have similar representations in the embedding space, thereby maintaining the local smoothness of the graph structure. By minimizing the differences in embedding representations between adjacent nodes, connected nodes become closer in the embedding space. This approach enhances the continuity of the learned embeddings while preserving the graph's topological structure.


(14) $$\begin{array}{l} L_{smoorh}\left(A,X\right)=\frac{1}{2n^{2}}\underset{i,j}{\sum }A_{ij}||x_{i}-x_{j}{||}^{2}=\frac{1}{n^{2}}trace\left(X^{T}LX\right) \end{array}$$

where the function *trace*(·) refers to the trace operation on matrices, *A* represents the adjacency matrix, *X* denotes the feature matrix, *L* is the Laplacian matrix, and *n* is the number of nodes.

Degree regularization loss focuses on balancing the distribution of node degrees, preventing excessive attention to nodes with either high or low degrees. By incorporating degree regularization, which considers the impact of node degrees, the learned representations gain greater universality and are less influenced by the degree of specific nodes.


(15) $$\begin{array}{l} L_{\text{degree}}\left(A\right)=-\frac{\beta }{n}1^{T}\log\left(A1\right) \end{array}$$

where *β* is a non-negative hyperparameter, and **1** represents a vector filled with 1.

Sparsity loss aims for sparsity in the embedding representations, resulting in many elements of the learned embeddings being zero. This reduces redundant information, making the embeddings more compact and efficient. By introducing sparsity loss, the efficiency of the expression of learned embeddings can be improved while retaining key information.


(16) $$\begin{array}{l} L_{sparsity}\left(A\right)=\frac{\theta }{n^{2}}||A{||}_{F}^{2} \end{array}$$

where *θ* is a non-negative hyperparameter, and $||\cdot {||}_{F}^{2}$ represents the *Frobenius* norm.

Considering both the prediction loss (Equation 13) and the graph regularization loss (Equation 17), the final joint loss (Equation 18) for IGGCN is obtained:


(17) $$\begin{array}{l} L_{G}=\omega L_{smooth}+L_{degree}+L_{sparsity} \end{array}$$

(18) $$\begin{array}{l} L=L_{pred}+L_{G} \end{array}$$

where *ω* is a weight parameter.

### 2.4 Iterative gated graph convolution network

**Figure 1:**
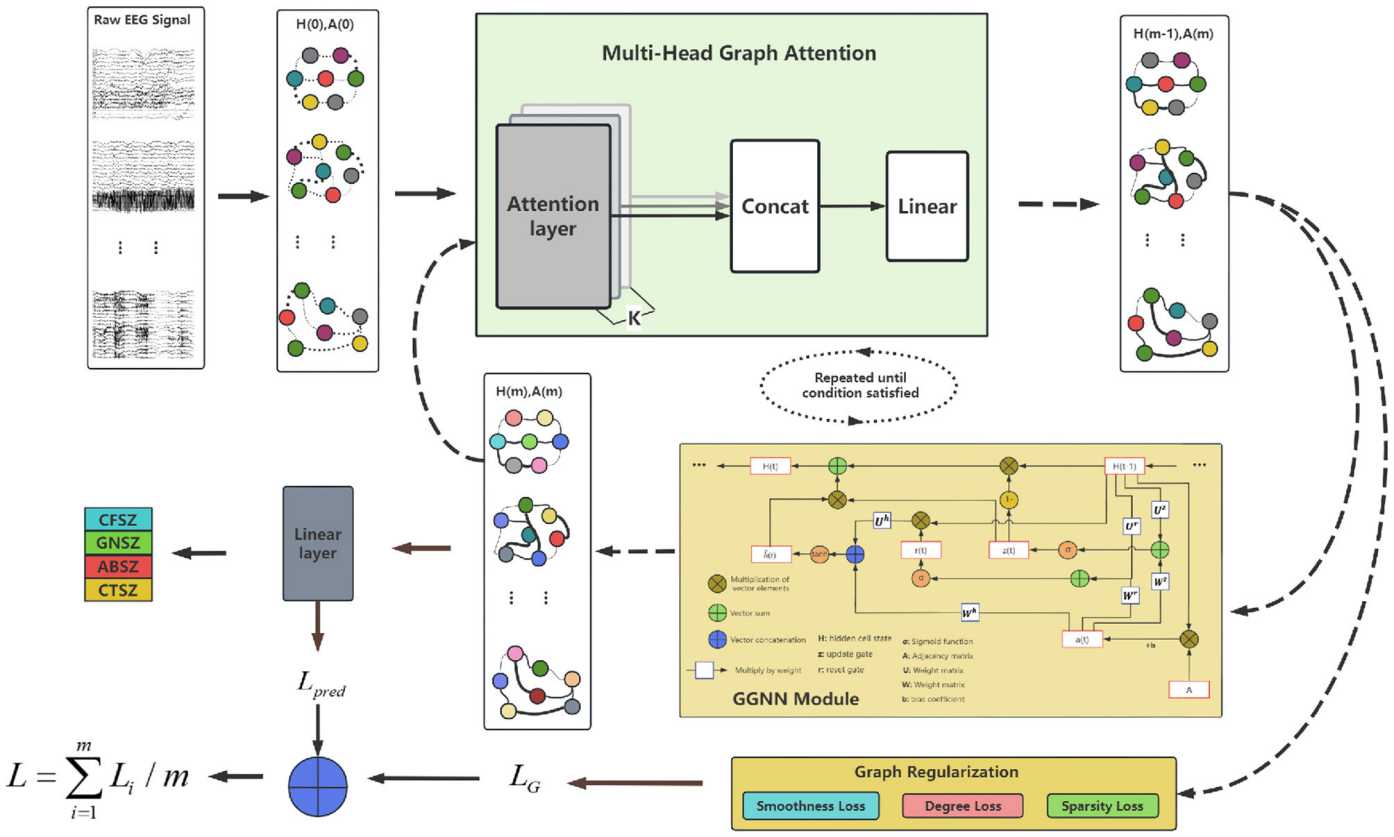
Overall framework of IGGCN model.

The IGGCN model is composed of three fundamental components: a multi-head attention module, a gated graph neural network module, and a graph regularization module, as illustrated in Figure 1.

Initially, the model constructs an initial graph structure *A*^(0)^ based on raw EEG data and calculates the feature representation for each EEG channel *H*^(0)^. The nodes in the graph represent EEG channels, while the node colors indicate their feature vectors. Then, *A*^(0)^ and *H*^(0)^ are input into the multi-head attention module to refine the graph structure. The updated graph structure *A*^(*m*)^ is subsequently passed through both the graph regularization module and the gated graph neural network module to update node features and obtain an updated node embedding *H*^(*m*)^.

During this process, the graph regularization module focuses on optimizing the graph structure, while the gated graph neural network module concentrates on classification tasks, leading to the generation of two distinct types of losses: the graph learning loss *L*_*G*_ and the prediction loss *L*_*pred*_. The total loss *L* is computed by integrating these two losses, which facilitates a comprehensive approach to model optimization.

The model undergoes several iterations of this process during training, refining both the graph structure and the feature representations with each cycle. This iterative approach ensures that the model progressively improves its ability to classify epilepsy EEG signals, leveraging the dynamic and complex relationships inherent in EEG data.

The complete algorithm for the IGGCN model, including all steps and processes involved, is detailed in Algorithm 1.

**Algorithm 1:**
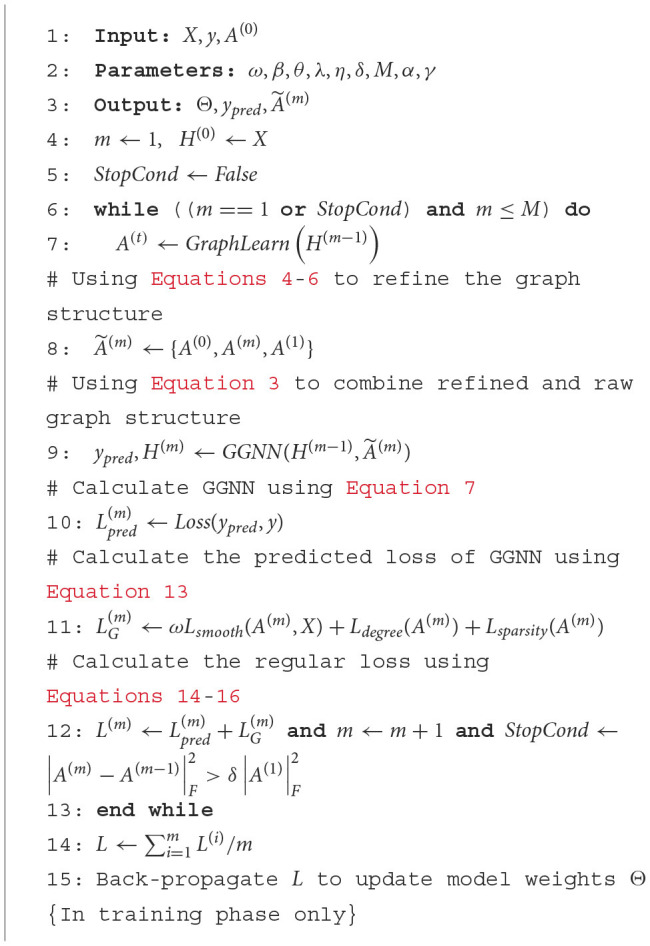
General Framework for IGGCN. ^*^Θ : A set of parameters *(W, U, b, etc.)* during training.

## 3 Dataset and preprocessing

### 3.1 Dataset

The dataset utilized in this study is derived from the TUSZ, version 1.5.2 (Obeid and Picone, 2016; Shah et al., 2018), which ranks among the most extensive epileptic EEG datasets globally available for research. This comprehensive database encompasses over 504 h of data, featuring 5,612 EEG recordings and 3,050 annotated clinical seizure records. It categorizes seizures into eight types: Focal Non-Specific Seizure (FNSZ), Generalized Non-Specific Seizure (GNSZ), Simple Partial Seizure (SPSZ), Complex Partial Seizure (CPSZ), Absence Seizure (ABSZ), Tonic Seizure (TNSZ), Tonic-Clonic Seizure (TCSZ), and Myoclonic Seizure (MYSZ).

Previous works (Fisher et al., 2017; Tang et al., 2021) reclassified the eight categories in the dataset into four categories. In this paper, we follow this approach as follows: firstly, since it is challenging to differentiate between FNSZ, SPSZ, and CPSZ based on EEG signals, these three seizure categories were combined into a single category named Combined Focal Non-Specific Seizure (CFSZ). Secondly, due to the small number of TNSZ, TCSZ, and MYSZ in the dataset, and given that TCSZ typically initiates with a tonic phase, these categories were merged into Combined Tonic Seizures (CTSZ). Thirdly, Myoclonic seizures are excluded because there are only two myoclonic seizures in the dataset. Consequently, four seizure types are finally generated: CFSZ, GNSZ, ABSZ and CTSZ.

In this paper, 19 EEG channels data in TUSZ were selected based on the international 10-20 lead system. These channels include “FP1”, “FP2”, “F3”, “F4”, “C3”, “C4”, “P3”, “P4”, “O1”, “O2”, “F7”, “F8”, “T3”, “T4”, “T5”, “T6”, “FZ”, “CZ” and “Z”. This selection is strategic to ensure comprehensive capture of brain activity across various regions, thereby facilitating a detailed analysis and classification of seizure types.

The original TUSZ dataset was carefully divided into training and test sets. To reduce the risk of inaccurate experimental results due to overfitting, we strategically excluded five patients from the test set (these five patients are present in both the training and test sets).

In addition, we randomly selected 24 patients from the training set to form a validation set. This set is used to evaluate the classification capability of the model after each training iteration. Table 1 shows the data distribution of train, validation and test sets.

**Table 1:** Data distribution of train set and test set.

	**CF seizures (% total)**	**GN seizures (% total)**	**AB seizures (% total)**	**CT seizures (% total)**	**Patients (% total)**
Train set	10,291 (55.44%)	3,779 (20.36%)	76 (0.41%)	327 (1.76%)	178 (79.11%)
Validation set	1,081 (5.82%)	461 (2.48%)	11 (0.06%)	32 (0.17%)	24 (10.67%)
Test set	1,415 (7.62%)	814 (4.39%)	79 (0.43%)	195 (1.05%)	23 (10.22%)

### 3.2 Data preprocessing

Previous work (Tang et al., 2021) extracted one 12-s slices of EEG signals from each seizure event, while discarding the rest of the data. While this approach helped to streamline the data processing process and focused on the signal characteristics of the critical time period, it may have ignored important information that other time periods during the seizure process contained. To make better use of the data and improve the comprehensiveness of the analysis, we split the complete EEG signal from each seizure event into multiple 12-s slices, rather than selecting a single 12-s slice.

**Figure 2:**
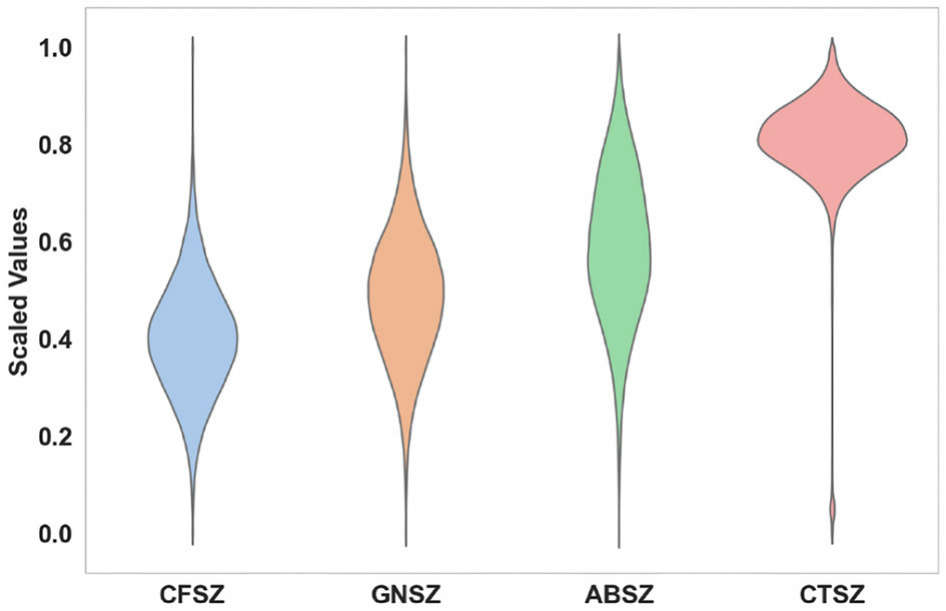
Log-amplitude characteristics of the four seizure classes.

Considering the significant correlation between seizures and frequency domain information (Tzallas et al., 2009), we first perform a fast Fourier transform (Brigham, 1988) on the original EEG signal to convert the signal from the time domain to the frequency domain. After extracting the positive frequency portion from the FFT result and applying a logarithmic operation, we perform Min-Max Scaling on these values. Figure 2 shows the distribution of features of the four distinct seizure classes of 19 EEG channels. We conduct an analysis of variance (ANOVA) (St and Wood, 1989) statistical test on the four classes. The *P*-value is close to zero, indicating that the characteristics of the four seizure classes are significantly different.

## 4 Experiments and results

### 4.1 Experiments settings

#### 4.1.1 Experimental environment

The experimental platform was Windows10, and the experimental environment was built with pytorch. The python version is 3.8.8 and the pytorch version is 2.0.1+cu117.

#### 4.1.2 Baseline models

We select several baseline models to compare against the IGGCN approach: Dense-CNN (Saab et al., 2020), LSTM (Hochreiter and Schmidhuber, 1997), CNN-LSTM (Ahmedt-Aristizabal et al., 2020), and DCRNN (Tang et al., 2021). Each of these baseline models undergoes the same preprocessing steps and is evaluated within the same experimental environment as the IGGCN. This consistency in preprocessing and experimental conditions ensures a fair comparison, allowing us to accurately assess the performance of IGGCN relative to state-of-the-art models in the field of epilepsy EEG classification.

#### 4.1.3 Hyperparameters

Each model trains using the Adam optimizer (Kingma and Ba, 2014). *hidden*_*size* represents the number of hidden neurons in the GGNN, with a value range of 64, 128, 256. *M* indicates the maximum number of iterations for iterative graph learning, with a value range of 5, 8, 12. *epoch* denotes the maximum number of training rounds, and 150 epochs are typically trained at an initial learning rate of 1e-3. *batch*_*size* indicates the number of batch samples (the value is 64). The parameters *ω*, *β* and *θ* are adjusted in the regularization of the graph, respectively. *K* is a positive integer representing the number of attention heads, taking values from the set 4, 8. *γ* is the balance parameter in Focal Loss. *num*_*node* is the number of nodes, corresponding to the number of EEG channels. Validation sets are used to search the best hyperparameters. All experiments were performed 5 times using different random seeds, and the results are presented as the mean plus the 95% confidence interval (Altan, 2022).

λ and *η* are weighted parameters for optimizing the graph structure, with values range of 0.2, 0.3, 0.4, 0.5. To achieve optimal performance with IGGCN, we conducted several experiments to identify the best parameter values. The experimental results are illustrated in Figure 5. IGGCN performs best when the values of parameters λ and *η* are 0.3 and 0.4, respectively. The optimal values of hyperparameters are shown in Table 2.

**Table 2:** Hyperparameter settings.

**Hyperparameter**	**Definition**	**Value**
*hidden*_*size*	Hidden cell size	256
*M*	Maximum iterations	10
*epoch*	Model training rounds	150
*batch*_*size*	Number of training batches	64
*ω*	Smoothing loss of balance parameter	0.5
*β*	Degree loss of balance parameter	0.01
*θ*	Sparse loss of balance parameter	0.3
*K*	Heads of attention	8
*γ*	Focal Loss modulation parameters	2
*num*_*node*	Number of electrical brain channels	19
λ	The weighted parameters of the initial and optimized graphs	0.3
*η*	The weighted parameters of the initial and optimized graphs	0.4

### 4.2 Results

#### 4.2.1 Performance comparison

Dense-CNN, LSTM, CNN-LSTM, as well as two DCRNN variants: correlation graphs (Corr-DCRNN) and distance graphs (Dist-DCRNN), alongside the IGGCN, are individually trained. We use Accuracy, Precision, Recall, and Weighted F1-Score for model evaluation. This approach ensures a detailed insight into each model's performance across various dimensions. The results of the test dataset are shown in Table 3. The results demonstrate the superior performance of IGGCN on all evaluated metrics, highlighting its superior accuracy and significant improvements in Recall and Weighted F1-Score, evidencing its robustness in multi-type epilepsy classification in EEG data.

**Table 3:** Classification results of four seizures categories under different models.

**Model**	**Accuracy (CI)**	**Precision (CI)**	**Recall (CI)**	**Weighted F1-score (CI)**
Dense-CNN	71.3% (69.6–73.1%)	68.2% (66.3–70.0%)	71.3% (69.6–73.1%)	63.8% (62.0–65.7%)
LSTM	69.5% (67.8–71.3%)	71.4% (69.6–73.1%)	69.5% (67.8–71.3%)	57.9% (56.1–59.7%)
CNN-LSTM	70.1% (68.4–71.9%)	69.3% (67.5–71.1%)	70.1% (68.4–71.9%)	59.9% (58.2–61.7%)
Corr-DCRNN	74.7% (73.1– 76.4%)	76.1% (74.5– 77.8%)	74.7% (73.1– 76.4%)	69.4% (67.7– 71.1%)
Dist-DCRNN	75.6% (73.9–77.2%)	74.1% (72.4–75.8%)	75.6% (73.9–77.2%)	72.0% (70.3–73.7%)
IGGCN	**91.8% (90.7–92.8%)**	**91.9% (90.8–93.0%)**	**91.8% (90.7–92.8%)**	**91.5% (90.5–92.6%)**

CI, 95% confidence intervals (lower-upper bound). The bold part is the optimal result of this evaluation indicator.

The experiment evaluates the classification effect of IGGCN by presenting the Area Under the Receiver Operating Characteristic (AUROC) values for each seizure category across all models, as shown in Table 4. The results demonstrate that IGGCN exhibits outstanding classification performance for all four seizure categories, showcasing its robust capability in distinguishing between different types of seizures.

**Table 4:** AUROC value of epileptic seizure classification under different models.

**Model**	**CFSZ (CI)**	**GNSZ (CI)**	**ABSZ (CI)**	**CTSZ (CI)**
Dense-CNN	69.9% (68.1–71.7%)	69.1% (67.3–70.9%)	79.4% (77.8–80.9%)	73.4% (71.7–75.1%)
LSTM	60.5% (58.5–62.3%)	53.9% (51.9–55.8%)	76.5% (74.9–78.2%)	72.9% (71.2–74.7%)
CNN-LSTM	64.3% (62.5–66.2%)	63.3% (61.4–65.2%)	70.9% (69.1–72.7%)	73.6% (71.9–75.4%)
Corr-DCRNN	78.6% (77.0–80.2%)	80.5% (78.9–82.0%)	85.6% (84.3–87.0%)	79.7% (78.2–81.3%)
Dist-DCRNN	81.4% (79.8–82.9%)	80.6% (79.0–82.1%)	93.2% (92.2–94.1%)	89.4% (88.2–90.6%)
IGGCN	**96.9% (96.2–97.6%)**	**97.0% (96.3–97.7%)**	**99.8% (99.6–99.9%)**	**98.8% (98.4–99.3%)**

CI, 95% confidence intervals (lower-upper bound). The bold part is the optimal result of this evaluation indicator.

**Figure 3:**
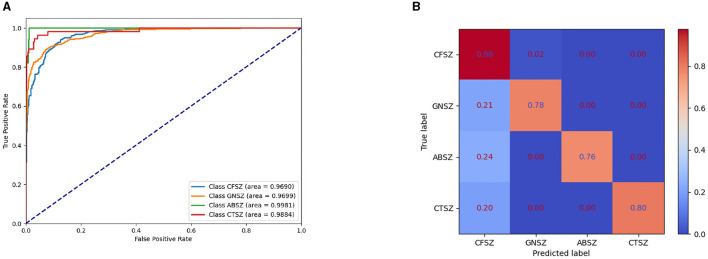
ROC curve and normalized confusion matrix of IGGCN model. **(A)** ROC curve for epilepsy classification. **(B)** Normalized confusion matrix.

Figure 3A shows the Receiver Operating Characteristic (ROC) curve for the IGGCN model, providing insight into its classification performance across four types of epilepsy seizure. The curve clearly indicates that the model achieves commendable classification results for all seizure types. Figure 3B shows the normalized confusion matrix of the IGGCN model, highlighting CFSZ (the seizure type with the largest number of training samples) as the easiest to distinguish. However, the other three categories are not as effectively classified, especially GNSZ, which is mostly misclassified as CFSZ. This visualization aids in understanding the model's performance nuances, particularly in areas where improvement is needed due to limited sample availability. It is worth noting that IGGCN has a relatively high prediction accuracy for GNSZ, and there is no situation where (Tang et al., 2021) misclassified a large number of GNSZ as CFSZ.

#### 4.2.2 Optimized graph

**Figure 4:**
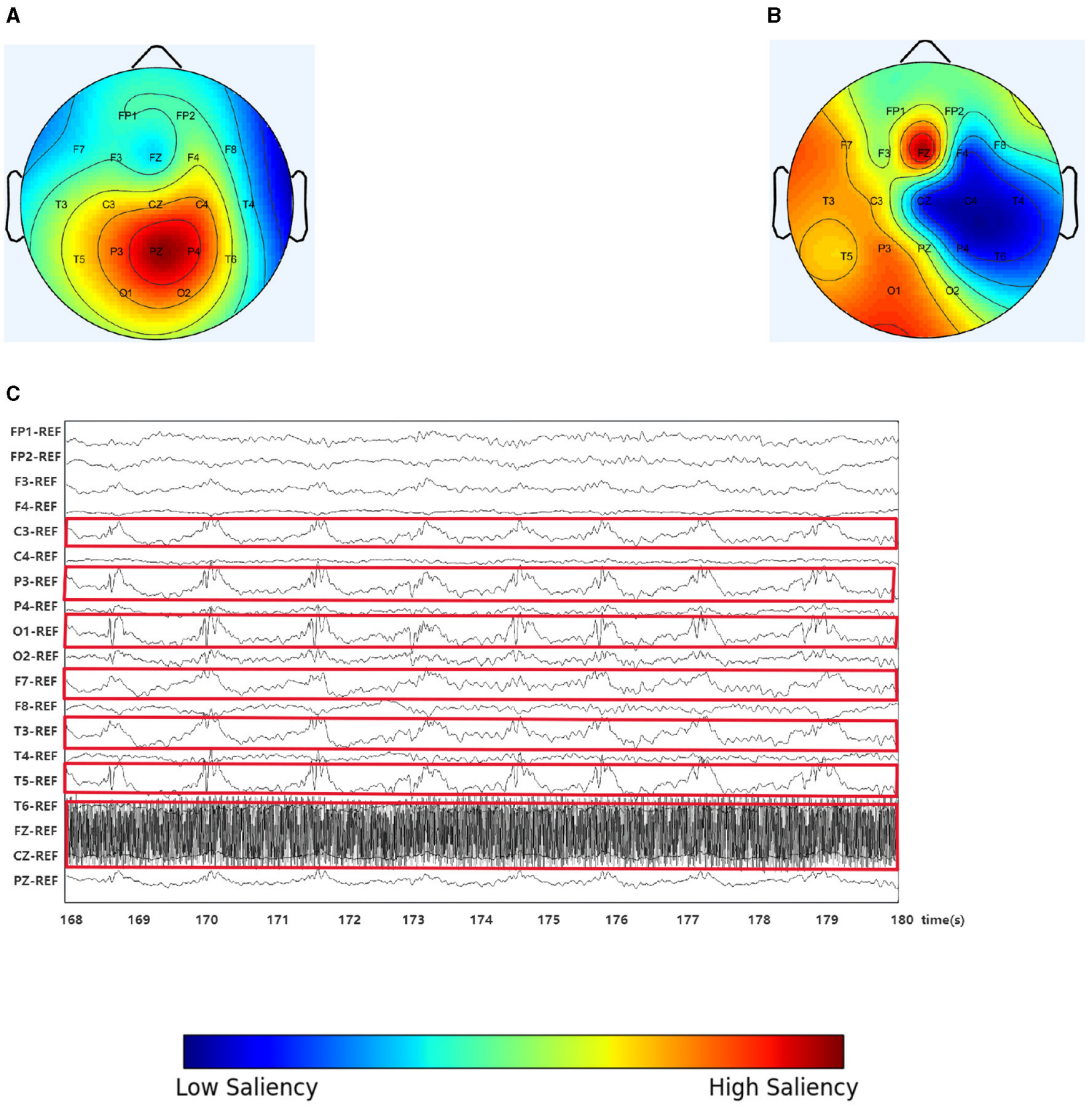
Spectral correlation graph, IGGCN model optimization graph and the EEG traces. **(A)** Spectral correlation graph. **(B)** Optimization graph. **(C)** The EEG traces.

In this study, we randomly selected an EEG slice from a patient, as shown in Figure 4. Figure 4A shows the original scalp topology based on the spectral correlation between channels (Wagh and Varatharajah, 2020), while Figure 4B shows the optimized topology of IGGCN, and Figure 4C displays the original EEG traces corresponding to the selected slice. IGGCN relies on the characteristics of each channel to optimize the topology. For channels exhibiting more significant epileptic seizures, the optimized topology enhances their attention; conversely, for channels with less pronounced seizures, the attention remains unchanged or decreases. From the original EEG traces (Figure 4C), it can be seen that the seizures are mainly concentrated in the left hemisphere of the brain (the related channels are marked with red boxes), including channels such as “C3”, “P3”, “O1”, “F7”, “T3” and “T5” which show strong seizure activity. Meanwhile, the “FZ” channel shows significant activity in the prefrontal area. However, in Figure 4A, “P3”, “PZ” and “P4” show the highest significance, which is inconsistent with the EEG traces in Figure 4C. This shows that the predefined graph cannot accurately reflect connections between EEG channels. The IGGCN model can optimize the input graph. After IGGCN optimization, as shown in Figure 4B, the significance of channels “C3”, “P3”, “O1”, “F7”, “T3”, “T5” and “FZ” is increased, while the significance of other channels is decreased. The optimized topology matches the original EEG traces, fully demonstrating the effectiveness of IGGCN in optimizing EEG topologies.

**Figure 5:**
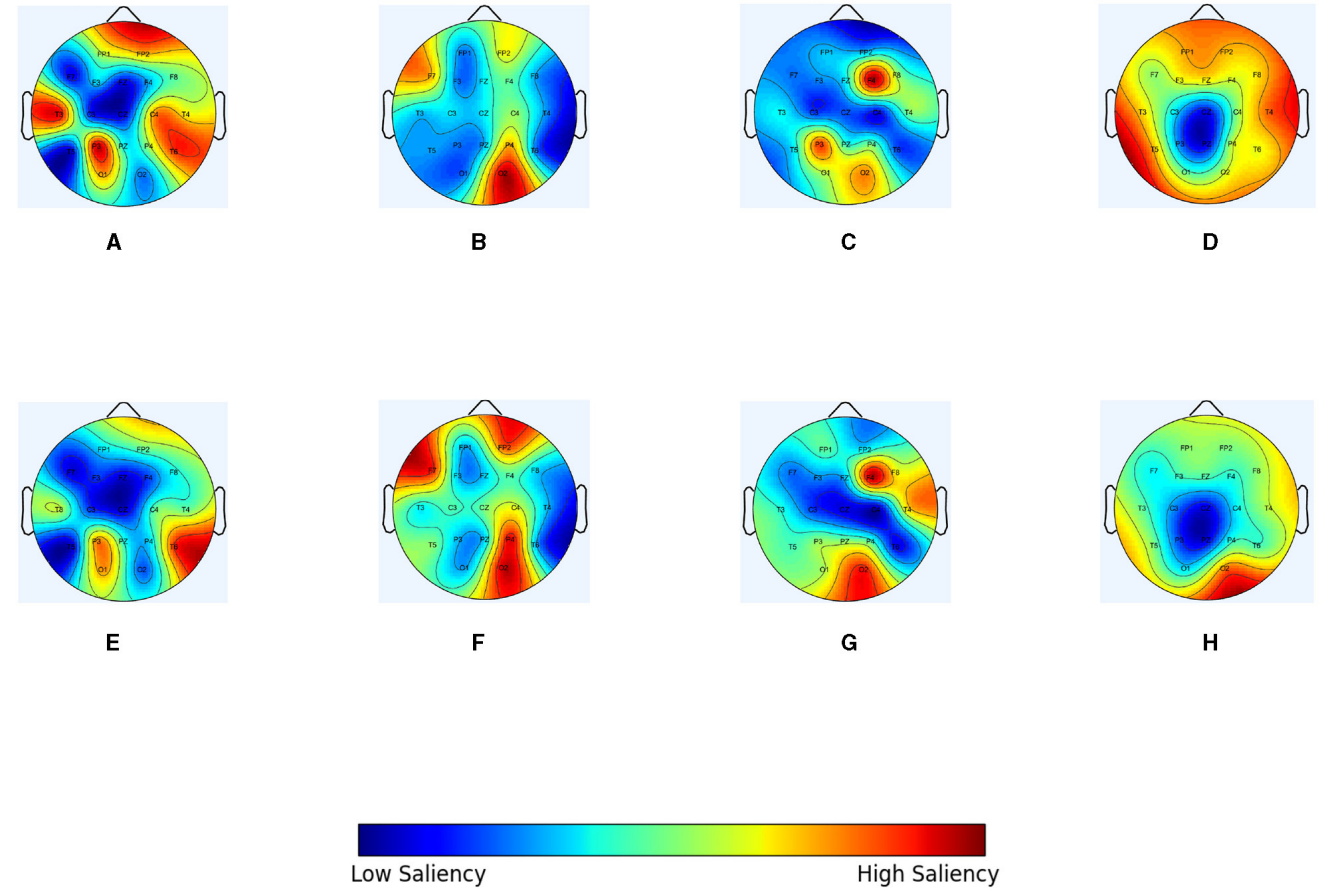
The topological optimization effect of IGGCN model on four types of epileptic seizure types. **(A)** Original graph of a specific slice for patient number 6904 (CFSZ). **(B)** Original graph of a specific slice for patient number 2380 (GNSZ). **(C)** Original graph of a specific slice for patient number 1413 (ABSZ). **(D)** Original graph of a specific slice for patient number 8444 (CTSZ). **(E)** Optimized graph of a specific slice for patient number 6904 (CFSZ). **(F)** Optimized graph of a specific slice for patient number 2380 (GNSZ). **(G)** Optimized graph of a specific slice for patient number 1413 (ABSZ). **(H)** Optimized graph of a specific slice for patient number 8444 (CTSZ).

In order to intuitively demonstrate the optimization effect of the IGGCN model on each type of epileptic seizure topology, epilepsy patients with seizure category CFSZ (ID “6904”), GNSZ (ID “2380”), ABSZ (ID “1413”), and CTSZ (ID “8444”) were selected to compare the topological structure of the original graph with the topological structure of the graph optimized by the model. The nodes represent EEG channels, and the weight is the mean of the edge weights. We use scalp topology maps to show the results in Figure 5, in which warmer colors indicate larger weight values. Figures 5A–D represent the spectral correlation topology, while Figures 5E–H display the optimized topology of IGGCN. It is evident that there are substantial differences between the predefined topologies before and after optimization; notably, the model focuses more on relevant channels corresponding to seizure types and less on irrelevant channels, thereby enhancing classification performance. For example, for patient “6904”, the significance of channels “FP2” and “T3” in the topology diagram before optimization is relatively high, because these two channels contribute little to the classification of this category, the model pays less attention to these two channels after optimization. Similarly, for patient “2380”, the IGGCN model has added attention to channels “FP2”, “F7,” and “P4”. The significance of “T4” and “O2” in “1413” patients is significantly increased, while the significance of “P3” is weakened. The topological structure of “8444” patients is the most obvious change, and the significance of most channels was reduced. However, the significance of the “O2” channel is significantly improved.

#### 4.2.3 Hyperparameter tuning

**Figure 6:**
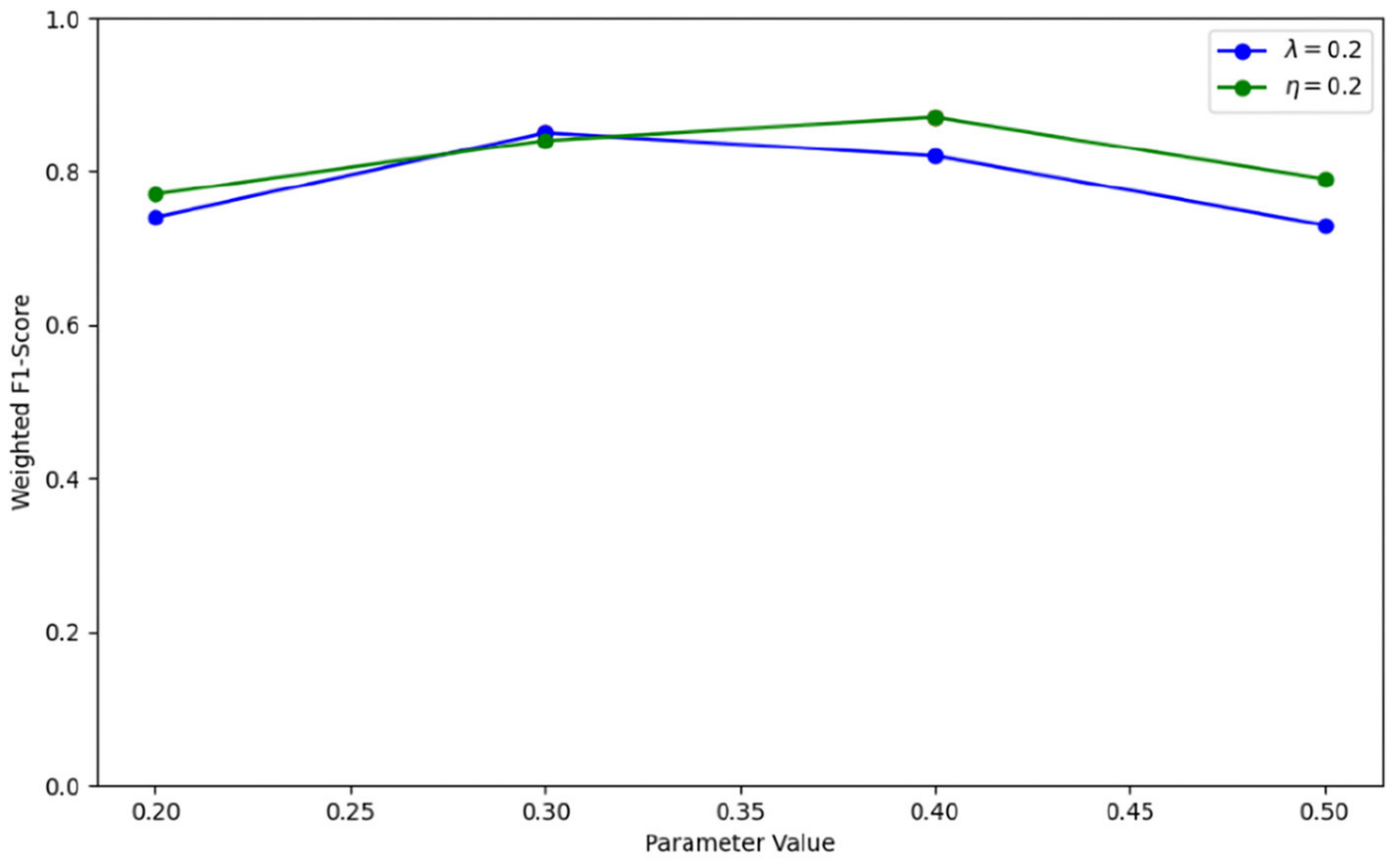
The impact of parameters λ and *η* on IGGCN.

In the iterative optimization diagram of IGGCN, parameters λ and *η* are crucial as they significantly impact the model's classification performance. To select the optimal values for these parameters, we set one parameter to 0.2 and vary the other within the range of 0.2, 0.3, 0.4, 0.5. The experimental results are shown in Figure 6. It can be seen that when parameter λ is 0.3 and parameter *η* is 0.4, the model achieves the best performance.

#### 4.2.4 Ablation experiment

To evaluate the impact of each component in IGGCN model, ablation studies are performed, and the results are detailed in Table 5. This investigation encompasses evaluating the baseline IDGL model, the IGGCN without the GGNN module, the IGGCN without the graph attention module, and the full IGGCN model. All models employ the Focal Loss function. The results show IGGCN' superior performance in the task of classifying four types of seizures, marking a notable enhancement across various metrics when juxtaposed with the baseline IDGL model. IGGCN, by integrating the strengths of both IDGL and GGNN models, emerges as better suited for seizure classification. The experimental results not only underline IGGCN's adeptness in seizure classification but also confirm the significant contributions of the GGNN module and graph attention mechanism in amplifying the model's overall effectiveness.

**Table 5:** Results of ablation experiment.

**Model**	**Accuracy (CI)**	**Precision (CI)**	**Recall (CI)**	**Weighted F1-score (CI)**
IDGL	69.7% (67.9–71.4%)	63.6% (61.7–65.4%)	69.7% (67.9–71.4%)	62.1% (60.8–63.4%)
IGGCN (w/o GGNN)	76.7% (75.0–78.3%)	77.3% (75.7–78.9%)	76.7% (75.0–78.3%)	72.8% (71.6–73.9%)
IGGCN (w/o Graph Attention)	75.9% (74.3–77.6%)	76.9% (75.3–78.6%)	75.9% (74.3–77.6%)	71.4% (70.2–72.6%)
IGGCN	**91.8% (90.7–92.8%)**	**91.9% (90.8–93.0%)**	**91.8% (90.7–92.8%)**	**91.5% (90.5–92.6%)**

CI, 95% confidence intervals (lower-upper bound). The bold part is the optimal result of this evaluation indicator.

To highlight the effectiveness of Focal Loss in enhancing model performance, a comparative study is conducted on the IGGCN model utilizing various loss functions, including Categorical Cross-Entropy Loss (Zhang and Sabuncu, 2018), Weighted Cross-Entropy Loss (Phan and Yamamoto, 2020), Generalized Dice Loss (Sudre et al., 2017), and Focal Loss. The findings, detailed in Table 6, underscore Focal Loss's outstanding contribution across several key metrics Accuracy, Precision, Recall, and Weighted F1-Score. Notably, Focal Loss achieves an Accuracy of 91.8%, Precision of 91.9%, Recall of 91.8%, and a Weighted F1-Score of 91.5%. These figures significantly surpassed the performance metrics recorded for the other loss functions. Focal Loss is designed to address class imbalances in classification problems by introducing dynamic weight adjustment mechanisms and modulation coefficients into the loss function, making the model more focused on samples that are difficult to classify correctly. This method not only enhances the model's ability to identify minority class samples but also ensures its easy implementation and low computational complexity through its concise formula design. The application of Focal Loss effectively addresses the issue of class imbalance and improves the overall performance of the IGGCN model in the task of epilepsy classification. In summary, owing to the significant performance enhancement provided by Focal Loss and its efficient strategy for tackling class imbalance, this study identifies it as the optimal predictive loss function for the IGGCN model.

**Table 6:** Results of using different loss functions.

**Model**	**Accuracy (CI)**	**Precision (CI)**	**Recall (CI)**	**Weighted F1-score (CI)**
Categorical cross-entropy loss	68.2% (66.3–70.1%)	61.4% (59.6–63.3%)	68.2% (66.3–70.1%)	62.2% (60.9–63.5%)
Weighted cross-entropy loss	71.1% (69.3–72.9%)	69.5% (67.7–71.3%)	71.1% (69.3–72.9%)	63.6% (62.3–64.9%)
Generalized dice loss	71.6% (69.8–73.3%)	71.1% (69.4–72.9%)	71.6% (69.8–73.3%)	63.9% (62.2–65.6%)
Focal loss	**91.8% (90.7–92.8%)**	**91.9% (90.8–93.0%)**	**91.8% (90.7–92.8%)**	**91.5% (90.5–92.6%)**

CI, 95% confidence intervals (lower-upper bound). The bold part is the optimal result of this evaluation indicator.

## 5 Discussion

Seizure classification is crucial for enhancing patient outcomes and medical operational efficiency. Although traditional machine learning techniques have provided valuable insights in some instances, they sometimes fall short of addressing the rich spatio-temporal complexity within EEG signals. The emergence of deep learning methods, such as CNN (Acharya et al., 2018; Zhou et al., 2018; Zhang Y. et al., 2019; O'Shea et al., 2020; Altan et al., 2021), LSTM (Tsiouris et al., 2018; Hu et al., 2020), CNN-LSTM (Shahbazi and Aghajan, 2018), and GCN (Zhang T. et al., 2019; Wagh and Varatharajah, 2020; Zhong et al., 2020; Tang et al., 2021; Raeisi et al., 2022), has marked a notable shift. CNN are designed for processing grid-like data, leveraging local connectivity and weight sharing to efficiently capture spatial features. However, their focus on grid-like data limits their ability to capture the irregular connections in EEG data, hindering their ability to fully capture the intricate interplay among multiple electrode signals collected from EEG recordings. LSTM are specialized for sequential data, using memory cells to retain long-term dependencies, but they struggle with the spatial dependencies inherent in EEG signals. GCN, on the other hand, excel at studying the intricate interplay among multiple electrode signals due to their strong processing capabilities for capturing the complex spatial dependencies and relational structures inherent in graph-structured data collected from EEG data. However, GCN also face challenges: First, the graph topology often relies on expert knowledge, which may not accurately reflect connections between EEG channels and varies significantly across patients, making it difficult to create a universal graph. Second, GCN struggle with memorizing long-term sequence information, which is essential for recognizing and utilizing long-term dependencies in EEG signals.

To address these issues, we propose IGGCN, a model designed to refine the graph topology through iterative learning, allowing for a more precise depiction of the intricate interplay within EEG signals. The GGNN module effectively captures the long-term dependencies among nodes in the EEG signal topology through a sophisticated gating mechanism. The results of ablation experiments result in Table 5 confirms the efficacy of iterative learning and GGNN module in seizure classification tasks. The results in Table 6 demonstrate the effectiveness of Focal Loss in addressing class imbalance in epilepsy classification tasks. The results of comparisons with other baseline models in Table 3 reveal that the IGGCN model significantly improves classification performance. These findings suggest that the IGGCN model offers an innovative and potent approach to the automated classification of epileptic seizures.

However, the IGGCN model still encounters certain issues. The confusion matrix analysis in Figure 3B shows that the CFSZ category achieves superior classification outcomes, likely because of its large proportion in the training dataset, which causes the model to focus more on this category. As a result, the classification performance of other categories, such as GNSZ, CTSZ, and ABSZ, has decreased. Moreover, all misclassified instances are incorrectly assigned to the CFSZ category, which has the highest number of samples. Although the IGGCN model incorporates Focal Loss to balance the model's attention across different seizure types and mitigate the impact of class imbalance, the efficacy of this approach still requires enhancement. We will explore additional, more effective strategies to address class imbalance in the future.

The IGGCN model combines graph learning and GGNN layers in each iterative step, which adds to the complexity of its training process. Experimental data reveals that the model encompasses 0.85M trainable parameters. Within an RTX 3080 GPU experimental setup, each training epoch consumes about 7 min, culminating in a total training duration of around 17 h. As depicted in Table 7, performance evaluations for all models were conducted using identical hardware configurations. In the future, we plan to light-weight the model architecture to reduce the number of model parameters, thereby improving the practicality of the model and making it more competitive in actual scenarios.

**Table 7:** Comparison of parameter number and running time of different models.

**Model**	**Parameter quantity**	**Duration epochs**
Dense-CNN	10.92 M	20-min
LSTM	0.50 M	5-min
CNN-LSTM	5.9 M	10-min
Corr-DCRNN	0.28 M	4-min
Dist-DCRNN	0.16 M	4-min
IGGCN	0.85 M	7-min

## 6 Conclusion and future work

EEG data, inherently non-Euclidean and irregular, poses unique challenges for the classification of epilepsy seizures. To address this, we introduce a novel approach termed IGGCN, which synergizes IDGL with GGNN to enhance seizure classification. By iteratively refining the EEG graph structure, IGGCN significantly improves classification outcomes. Demonstrated on a substantial public dataset TUSZ, the method achieves remarkable results, notably in Recall and Weighted F1-Score, which stand at 91.8 and 91.5% respectively. This marks a considerable advancement in classifying less common epilepsy types. To mitigate the effects of dataset imbalance, the Focal Loss function is employed, boosting Accuracy in minority classes. IGGCN offer a promising tool for clinical epilepsy diagnosis, providing critical insights to clinicians and easing their diagnostic workload. Beyond EEG data, the methodology introduced here presents a versatile framework applicable to a range of research fields utilizing graph-based data representations.

In the future, we will explore more effective ways to address class imbalances while making lightweight improvements to the IGGCN architecture to improve the model's usefulness. These advances will provide healthcare professionals with more efficient and precise diagnostic insights, enabling the implementation of personalized treatment strategies.

## Data Availability

The Temple University Hospital EEG Seizure Corpus used in this study is publicly available at https://isip.piconepress.com/projects/tuh_eeg. In addition, we provide data processing and modeling tools for the TUSZ datasets in this study at https://github.com/freesui1984/IGGCN_.
